# Biological Stability of Drinking Water: Controlling Factors, Methods, and Challenges

**DOI:** 10.3389/fmicb.2016.00045

**Published:** 2016-02-01

**Authors:** Emmanuelle I. Prest, Frederik Hammes, Mark C. M. van Loosdrecht, Johannes S. Vrouwenvelder

**Affiliations:** ^1^Environmental Biotechnology Group, Department of Biotechnology, Faculty of Applied Sciences, Delft University of TechnologyDelft, Netherlands; ^2^Department of Environmental Microbiology, Eawag – Swiss Federal Institute of Aquatic Science and TechnologyDübendorf, Switzerland; ^3^Division of Biological and Environmental Science and Engineering, Water Desalination and Reuse Center, King Abdullah University of Science and TechnologyThuwal, Saudi Arabia; ^4^Wetsus – European Centre of Excellence for Sustainable Water TechnologyLeeuwarden, Netherlands

**Keywords:** bacterial competition, water treatment optimization, water distribution conditions, flow cytometry, bacterial growth potential

## Abstract

Biological stability of drinking water refers to the concept of providing consumers with drinking water of same microbial quality at the tap as produced at the water treatment facility. However, uncontrolled growth of bacteria can occur during distribution in water mains and premise plumbing, and can lead to hygienic (e.g., development of opportunistic pathogens), aesthetic (e.g., deterioration of taste, odor, color) or operational (e.g., fouling or biocorrosion of pipes) problems. Drinking water contains diverse microorganisms competing for limited available nutrients for growth. Bacterial growth and interactions are regulated by factors, such as (i) type and concentration of available organic and inorganic nutrients, (ii) type and concentration of residual disinfectant, (iii) presence of predators, such as protozoa and invertebrates, (iv) environmental conditions, such as water temperature, and (v) spatial location of microorganisms (bulk water, sediment, or biofilm). Water treatment and distribution conditions in water mains and premise plumbing affect each of these factors and shape bacterial community characteristics (abundance, composition, viability) in distribution systems. Improved understanding of bacterial interactions in distribution systems and of environmental conditions impact is needed for better control of bacterial communities during drinking water production and distribution. This article reviews (i) existing knowledge on biological stability controlling factors and (ii) how these factors are affected by drinking water production and distribution conditions. In addition, (iii) the concept of biological stability is discussed in light of experience with well-established and new analytical methods, enabling high throughput analysis and in-depth characterization of bacterial communities in drinking water. We discussed, how knowledge gained from novel techniques will improve design and monitoring of water treatment and distribution systems in order to maintain good drinking water microbial quality up to consumer’s tap. A new definition and methodological approach for biological stability is proposed.

## Introduction

The World Health Organization [WHO] (2006) stated that “Water entering the distribution system must be microbiologically safe and ideally should also be biologically stable." There is general consensus that the term ‘biological stability’ in this context refers to the concept of maintaining microbial water quality from the point of drinking water production up to the point of consumption (Rittmann and Snoeyink, 1984; van der Kooij, 2000). Unwanted changes in microbial quality of drinking water can have adverse effects on distribution system and consumers. For example, during distribution, excessive growth of bacteria can lead to deterioration of drinking water quality in terms of safety (e.g., pathogens), consumer’s perception (e.g., discolouration) and operational aspects (e.g., biocorrosion; Szewzyk et al., 2000; Vreeburg et al., 2004; Sun et al., 2014). Changes in microbial water quality are a result of complex interactions between various organisms (bacteria, viruses, protozoa, higher organisms) regulated by: access to available growth-limiting nutrients, response to environmental conditions, such as water temperature, presence of potential residual disinfectant and other inhibitory substances, attachment of bacteria to pipe walls, particle deposition, sediment re-suspension and biofilm formation. The aim behind the concept of biological stability is that minimum change in water quality is occurring during drinking water distribution, or at least not to a degree that affects consumer’s safety or aesthetic perception or cause technical failure. To achieve this and limit bacterial growth during transport, drinking water is distributed in numerous countries with disinfectant residuals, using different substances (e.g., free chlorine, chlorine dioxide, monochloramine) at varying concentrations (Servais et al., 1995; LeChevallier et al., 1996; Gillespie et al., 2014). Adverse health effects of disinfection by-products and altered water taste have, however, led several countries to opt for water distribution without the addition of disinfectant to the produced drinking water (Vital et al., 2012a; Lautenschlager et al., 2013; Prest et al., 2014). In the latter case, minimum change in water quality is achieved in the first place by controlling the water quality with extensive water treatment strategy, and secondly by distributing water in well-maintained piping systems (van der Kooij, 2003).

A number of methods to assess bacterial growth-supporting properties of water have been developed during the last three decades to provide support to water utilities for the improvement of water treatment and distribution conditions in the context of biological stability (van der Kooij et al., 1982; Servais et al., 1989). In addition, several studies have addressed the effect of individual distribution-related factors on changes in drinking water quality (Supplementary Table S1). Concomitantly with methodological and experimental advances in this field, definitions of biological stability, as well as methods and approaches to address the concept have evolved (Rittmann and Snoeyink, 1984; Sibille, 1998; van der Kooij, 2000, 2003; Lautenschlager et al., 2013). In recent years, high-throughput analytical and molecular methods have emerged, enabling detailed characterization of bacterial communities in water (for review, see Douterelo et al., 2014), and distribution networks have been examined with an increasingly ecology-oriented approach, in which interactions between organisms are investigated (Berry et al., 2006; Proctor and Hammes, 2015).

The objective of the present paper is to review existing knowledge, future challenges and emerging ideas that aim to achieve and monitor biological stability of drinking water in full-scale distribution systems. We examine existing definitions and approaches to address biological stability, highlight information gaps and propose an updated definition and a strategy for assessment and monitoring of biological stability.

## Problems Associated with Bacterial Growth in Drinking Water Distribution Systems

The presence of bacteria in drinking water *per se* is not an issue, as long as no pathogenic organisms are present: there are bacteria in drinking water, even in relatively high numbers (10^3^ to 10^6^ cells/mL), without consequences on human health (Hoefel et al., 2005; Hammes et al., 2008; Vital et al., 2012a). However, unwanted and/or excessive bacterial growth in drinking water distribution systems can cause deterioration of microbial water quality during storage and transport. Firstly, a number of hygienically relevant opportunistic pathogens, such as *Pseudomonas aeruginosa, Legionella pneumophila, Mycobacteria, Aeromonas hydrophila, Klebsiella pneumoniae*, and *Campylobacter* have the capacity to grow at low nutrient concentrations in drinking water distribution systems and/or in households (Szewzyk et al., 2000; Flemming et al., 2002; Vital et al., 2008, 2012b; Wang et al., 2013a). In addition to bacterial species, certain protozoa have pathogenic properties (e.g., *Acanthamoeba, Cryptosporidium, Giardia lamblia*), or act as hosts for pathogenic bacteria such as *Legionella pneumophila* (Bichai et al., 2008; Thomas and Ashbolt, 2011; Wang et al., 2013a), while enteric viruses were recognized to cause water-born gastrointestinal or other viral illness (e.g., noroviruses, Hepatitis A virus; Wingender and Flemming, 2011). In Europe, 86 drinking waterborne disease outbreaks were reported in the period 1990–2005, of which 19 were identified as being caused at distribution level. However, the majority of distribution outbreaks were caused by external contamination, and only four outbreaks were attributed to growth of microorganisms in biofilms, stagnating water and/or re-suspension during distribution system flushing (Risebro et al., 2007). In the USA, 32 outbreaks were reported in the period 2011–2012, out of which 21 were related to Legionella (Beer et al., 2015). Secondly, deterioration of aesthetic aspects of drinking water, such as taste, odor, and color represents up to 80% of consumer complaints to water utilities (Polychronopolous et al., 2003; Vreeburg and Boxall, 2007). Turbid or discolored water is the result of particles in suspension (Vreeburg et al., 2004), which can originate from excessive growth of non-pathogenic bacteria within drinking water distribution systems, attached to particles, sediments or biofilms. These can be re-suspended in the water and cause yellowish colored water (Gauthier et al., 1999; Vreeburg and Boxall, 2007). Red or black colored water can be the consequence of iron particles and manganese precipitates (Sly et al., 1990; Seth et al., 2004), which can be partially produced by bio-corrosion of iron pipes (Sun et al., 2014) or manganese oxidizing or reducing organisms (Cerrato et al., 2010). Moreover, specific bacteria produce molecules affecting taste and odor of water. Typical examples are actinomycetes, which produce geosmin, responsible for an earthy-muddy water taste (Srinavasan and Sorial, 2011), and bacteria involved in the sulfur cycle (e.g., sulfate reducing or oxidizing bacteria) that can promote a sulfur-based odor (Scott and Pepper, 2010). Besides, yeast, fungi, and algae have also been recorded in drinking water and some of these organisms have been associated with taste and odor complaints (Block et al., 1993; Sibille et al., 1998; van der Wielen and van der Kooij, 2013). In addition, bacteria represent the start of a trophic chain, and high bacterial numbers would result in the occurrence of protozoa and of invertebrates such as crustaceans (e.g., *Asellidae*), worms (e.g., annelida) or snails (e.g., mollusca) in distribution systems (van Lieverloo et al., 2002a; Christensen et al., 2011). The presence of invertebrates and particularly of the large *Asellus aquaticus* (2–10 mm long; Christensen et al., 2011) in household taps is negatively perceived by consumers (van Lieverloo et al., 2002a). Thirdly, operational problems were related with bacterial activity, such as fouling of concrete pipes due to growth of bacteria to high numbers in the form of a biofilm (Flemming, 2002; Allion et al., 2011), or biocorrosion of cast-iron pipes promoted by, e.g., sulfate-reducers and iron-oxidizers (Lee et al., 1980; Emde et al., 1992; Sun et al., 2014). The replacement of damaged distribution pipes related to microbial processes represents one major financial investment for water utilities. Finally, non-compliance with regulatory guidelines on, e.g., HPC or *Aeromonas* counts (Anonymous, 1998; Waterleidingbesluit, 2001; Sartory, 2004) can be caused by growth of culturable heterotrophic bacteria or by increased bacterial culturability as a result of favorable conditions. For example, HPC measured in drinking water sampled at long residence times in a distribution system in Germany during a warm summer (water temperatures above 20°C) were excessively high, sometimes exceeding the German guideline value of 100 CFU/mL, while HPC values in the treatment eﬄuent were below 5 CFU/mL (Uhl and Schaule, 2004). Similarly, Lautenschlager et al. (2010) showed that HPC in water stagnated in premise plumbing of six out of 10 studied houses were higher than the recommended HPC value in Switzerland (300 CFU/mL), which was the result of increased HPC numbers during stagnation (up to 580-fold higher than in flushed tap water). Achieving biological stability and providing good drinking quality water to consumers require therefore not only to produce clean and safe water, but also to limit changes in the bacterial community during drinking water distribution that would lead to uncontrolled growth up to high bacterial cell numbers and to the occurrence of unwanted microorganisms.

## A Deeper Look into Microbial Dynamics in Drinking Water

**FIGURE 1:**
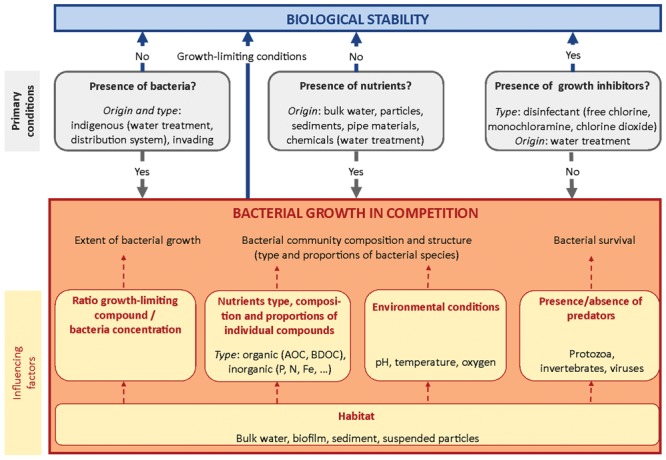
**Overview of primary conditions for bacterial growth and influencing factors of bacterial competition processes**.

In this section, factors affecting bacterial growth in drinking water are reviewed. Each factor is examined in view of its relevance for achieving biological stability, i.e., of its influence on shaping and/or modifying the bacterial community characteristics (bacterial abundance, viability, and community composition). An overview of primary conditions for bacterial growth and influencing factors on bacterial competition processes is given in **Figure 1**. Factors related to drinking water distribution conditions and influencing bacterial growth kinetics, such as temperature and time are discussed in Section “Factors Influencing Biological Stability in Distribution Networks.”

### Effect of Nutrient Concentration and Composition

**FIGURE 2:**
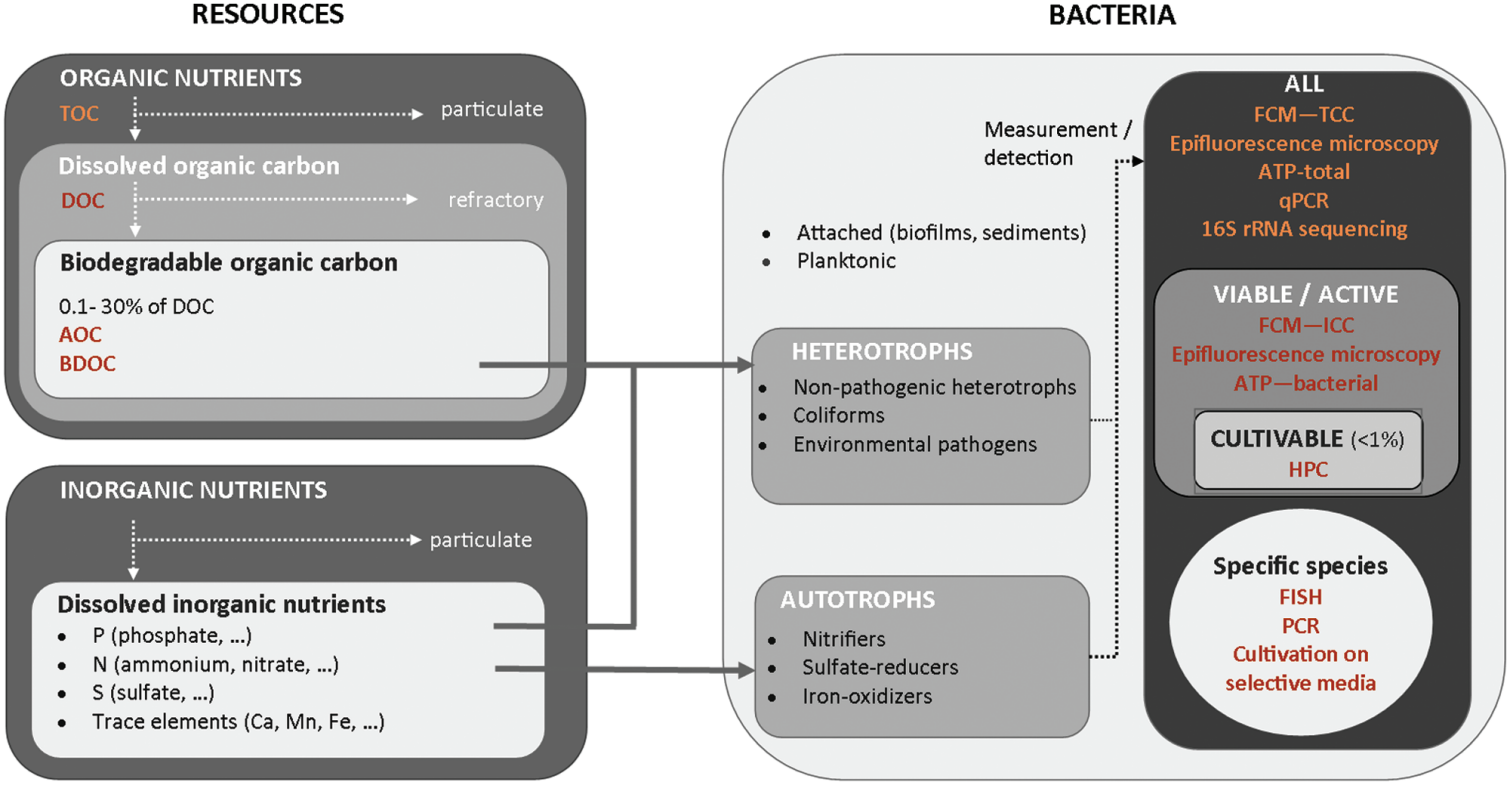
**Overview of resources available for different types of bacteria and of characterization methods of organic nutrients and bacterial communities in water**.

The composition and concentrations of individual substrates in drinking water are inherently related to biological stability, by limiting or promoting bacterial growth in water. In the first place, concentrations of available organic and inorganic nutrients govern the extent of bacterial growth (**Figure 2**). Heterotrophic organisms constitute the majority of bacteria in drinking water, and draw their energy from degradation of organic carbon compounds. Due to bacterial elemental composition (ratio C:N:P), organic carbon is most often the growth limiting compound and thus is particularly important for biological stability. BOM comprises a broad spectrum of different organic carbon compounds ranging from simple organic acids and sugars to complex polymeric substances, such as humic compounds (Münster, 1993; Schmidt et al., 1998). Only a fraction of the DOC can be utilized by bacteria as energy source for growth. Concentrations of available organic substrate typically range between 1 and 300 μg C/L when estimated by AOC methods (typically 0.1–10% of DOC) or range between 40 and 800 μg C/L when estimated by BDOC methods (1–30% of DOC; data compiled from references listed in Supplementary Table S1). Typical yield values for heterotrophic bacteria are between 4.6 × 10^6^ – 20 × 10^6^ cells/μg C (van der Kooij and Hijnen, 1985a; Hammes and Egli, 2005), which implies that an organic carbon concentration as low as 1 μg C/L is sufficient to promote the growth of 10^3^–10^4^ cells/mL (van der Kooij et al., 1980, 1982; van der Kooij and Hijnen, 1985b; Vital et al., 2012a). In the context of regulatory guidelines for HPC, typically in the range of 10^2^–10^3^ cells/mL, producing stable water is therefore challenging. Inorganic nutrients such as phosphorus, nitrogen or trace elements (iron, magnesium, copper, potassium…), are also required for heterotrophic growth, though in considerable smaller amounts than organic carbon (Ihssen and Egli, 2004). Very low concentrations in any essential inorganic compounds will result in heterotrophic bacterial growth limitation, as observed in waters with highly elevated organic carbon concentrations (Miettinen et al., 1997). Studies have, however, essentially focussed on organic carbon limitations so far and it is still unclear whether bacterial growth limitations in inorganic elements, including phosphate limitations, but also other elements, are frequent in drinking water systems.

While concentrations of individual substrates present in water define the growth-limiting substrate and control the extent of bacterial growth, the type of individual organic and inorganic substrates determines the type of organisms present in water. A typical example is the presence of methane-oxidizing bacteria in deep ground waters containing high concentrations of methane (de Vet et al., 2009; Lin et al., 2012). Though it is generally accepted that heterotrophic bacteria constitute the large majority of bacteria found in drinking water, presence of autotrophic organisms such as nitrifying, sulfate-reducing or iron-oxidizing bacteria has also been recorded in different drinking water systems (Rittmann and Snoeyink, 1984; Pepper et al., 2004). For example, ammonium oxidizing bacteria such as *Nitrosomonas* and *Nitrospira* are found in treated deep-ground waters rich in ammonium (de Vet et al., 2009), while sulfate-reducers (e.g., *Desulfovibrio* and *Desulfotomaculum*) and iron-oxidizers (e.g., *Gallionella, Leptothrix*, and *Sphaerotilus*) were associated with microbially induced corrosion processes (Emde et al., 1992; Sun et al., 2014). Dosage of monochloramine as residual disinfectant during drinking water transport was also shown to cause growth of ammonium oxidizing (e.g., from genus *Nitrosomonas*) or nitrite oxidizing bacteria (Wolfe et al., 1990; Lipponen et al., 2002). Clear data are lacking on the contribution of autotrophic growth in the total bacterial production and in the occurrence of aesthetic or operational related problems. Insights in functions of specific bacterial species in the water eco-system, and compounds and conditions required for their development would be a major step forward in the understanding of controlling factors of drinking water biological stability.

Besides type and concentrations of available substrates, composition and proportions of individual organic and inorganic compounds are essential parameters in the competition processes regulating bacterial growth, and are therefore essential to the concept of biological stability. Competition is a complex interplay between bacterial species, controlled by nutrient composition and proportion in water, physico-chemical parameters, such as water temperature or pH, and specific kinetic capabilities of individual species (**Figure 1**). As discussed above, drinking water contains numerous different nutrients at very low concentrations of individual compounds (Schmidt et al., 1998; Sibille, 1998; Wong et al., 2002). In such environment, bacteria are able to use simultaneously several nutrients for growth (Ihssen and Egli, 2004; Egli, 2010). The composition and concentration of nutrients defines an ecological niche, in which bacteria that have an overlap in substrate utilization spectrum will compete for available substrate (Hansen and Hubbell, 1980; Fredrickson and Stephanopoulos, 1981; Vital et al., 2012b). Therefore, composition and proportions of individual organic and inorganic compounds shape the bacterial community composition and structure, which would be affected by any disturbance in the nutrient pool (Gottschal et al., 1979). The complex nutrient composition in drinking water typically results in the presence of a large diversity in autochthonous bacterial species (Pinto et al., 2012; Yin et al., 2013; Liu et al., 2014), well adapted to survival and proliferation in oligotrophic environments. Bacterial communities with high richness and evenness have been shown to be potentially more resistant against growth of intrusive bacterial species and against environmental stress (Wittebolle et al., 2009; De Roy et al., 2013; van Nevel et al., 2013). One explanation may be the broad substrate utilization spectrum and the large range of functionality and metabolisms covered by bacteria. Based on these observations, one could argue that a drinking water containing a highly diverse bacterial community with high evenness would have a higher chance to remain stable during water distribution where conditions are changing (cf. details in section “Biological Stability in Drinking Water: Implications for Treatment and Distribution”). The role of complex bacterial competition processes for nutrients in drinking water and of bacterial diversity, richness and evenness for biological stability requires further research.

### Effect of Growth-Inhibiting Substances

The question of applying a disinfectant residual in water is central in the context of biological stability. Increased bacterial abundance in water has been observed when a residual disinfectant is partially or fully depleted in drinking water distribution systems (Servais et al., 1995; Nescerecka et al., 2014), due to reaction with bacterial cells, NOM, particles, sediments, and biofilms (Rossman et al., 1994; Gauthier et al., 1999; Campos and Harmant, 2002). Disinfectant threshold concentrations for bulk bacterial growth to occur are dependent on water quality and type of disinfectant applied. For example, LeChevallier et al. (1996) reported the occurrence of high numbers of bacteria of the coliform group in systems maintaining free chlorine concentrations below 0.2 mg/L and monochloramine concentrations below 0.5 mg/L, when AOC concentrations were above 100 μg/L. More recently, Gillespie et al. (2014) showed that drinking water distribution areas with free-chlorine concentrations below 0.5 mg/L were related to higher intact bacterial cell concentrations in bulk water than for areas with higher disinfectant concentrations. Moreover, biofilm development cannot be avoided at disinfectant concentrations used in drinking water distribution systems (LeChevallier et al., 1987; Revetta et al., 2013; Wang et al., 2014).

**FIGURE 3:**
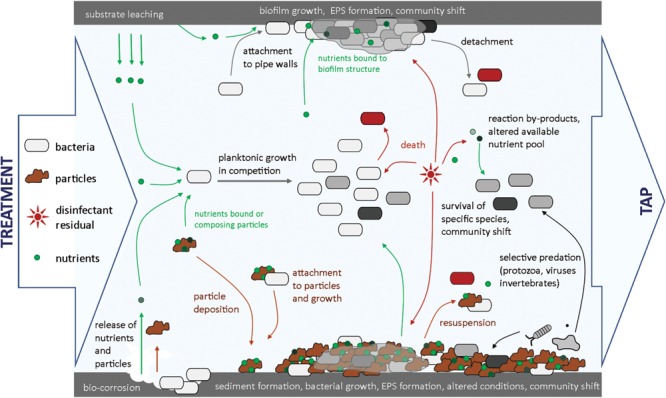
**Overview of microbial dynamics in a distribution pipe section.** Influences of pipe material, hydraulics, residual disinfectant, and bacterial predators on bacterial growth and community shifts are highlighted.

Addition and depletion of disinfectants in water has been shown to influence bacterial community composition and structure. Shifts in bacterial community and lower bacterial diversity were found in various systems after chlorination (Norton and LeChevallier, 2000; Roeder et al., 2010). Such shifts can be due to different resistance to chlorine of different bacterial species (Knochel, 1991; Abu-Shkara et al., 1998; Chiao et al., 2014), resulting in partial disappearance of the bacterial community after chlorine addition (**Figure 3**). Chiao et al. (2014) have shown with laboratory experiments that genera, such as *Dechloromonas* and *Acidovorax* were most sensitive to monochloramine compared to highly resistant genera, such as *Geobacter* or *Legionella*. One possible consequence is that a lower variety of substrate is covered by the remaining bacterial community, meaning that more niches are available for bacterial growth once disinfectant is depleted. The available substrate pool can also be modified by reaction of residual disinfectants with NOM, resulting in formation of low molecular weight assimilable organic carbon compounds (Reckhow et al., 1990; Fass et al., 2003), which may subsequently cause a shift in bacterial community composition. Disinfectants such as monochloramine have also been shown to support the growth of specific bacteria, in this case nitrifying bacteria in distribution systems (Lipponen et al., 2002).

### Effect of Other Microorganisms in Drinking Water Distribution Systems

The importance of bacterial growth control by other organisms than bacteria (e.g., protozoa, invertebrates, viruses) present in drinking water distribution systems is still unclear. Bacteria represent the start of a trophic chain in drinking water, and are subject to predation by organisms, such as protozoa, which in turn are targets for invertebrates (**Figure 3**) (Sibille, 1998; van Lieverloo et al., 2002a). Selective grazing by protozoa is likely to affect bacterial abundance and community composition (Wang et al., 2013a). The presence of protozoa in drinking water systems has been reported in concentrations ranging from 5 × 10^4^ to 7 × 10^5^ protozoa/L (Sibille, 1998), and has been linked to the presence of bacteria (Servais et al., 1995). Moreover, both protozoa and invertebrates excrete inorganic and organic nutrients, that are utilizable by bacteria and therefore modify the pool of available nutrients for bacterial growth (Sherr and Sherr, 2002; Wang et al., 2013a).

### Location of Bacterial Cells: In Water, Sediment, and Biofilm

Bacteria attached to surfaces, such as pipe surfaces, deposited particles/sediments, and suspended particles, grow in a significantly different environment in comparison with conditions in bulk water (**Figure 3**), which has considerable influence on abundance, growth rates, and composition of the bacterial community (Boe-Hansen et al., 2002a; Liu et al., 2014).

#### Characteristics of Drinking Water Biofilms

Biofilms are aggregates of microbial cells, usually accumulated at a solid–liquid interface and encased in a matrix of highly hydrated EPS (Flemming and Wingender, 2010). Biofilms are initiated by adsorption of bacterial cells to a surface, followed by production of EPS by attached cells, and bacterial proliferation within the formed biofilm (**Figure 3**). Biofilm cell density in drinking water distribution systems can vary significantly with cell numbers in the range of 10^4^ to 10^8^ cells/cm^2^, and with the amount of active biomass, as measured with ATP concentrations in the range of 10^2^ to 10^4^ pg ATP/cm^2^ (Boe-Hansen et al., 2002b; Wingender and Flemming, 2004; Långmark et al., 2005; Liu et al., 2014). The EPS structure offers a protective environment against disinfectant residuals and against grazing organisms, and binds organic and inorganic compounds (LeChevallier et al., 1988; Flemming and Wingender, 2010). Due to extracellular enzymes in the EPS, biofilm bacteria can utilize complex organic substrates, such as humic acids that are not easily biodegradable and usually not used by bulk water bacteria (Camper, 2004; Flemming and Wingender, 2010). Availability of additional nutrients creates new ecological niches and thus enables growth of different microorganisms than present in bulk water. Liu et al. (2014) reported that about 12% of the total bacteria (OTUs) found in biofilms were not shared with the bulk water. Shift in bacterial community between suspended and attached phases are furthermore influenced by specific pipe materials applied in drinking water distribution networks (see details in section “Biological Stability in Drinking Water: Implications for Treatment and Distribution”). Studies have shown that young biofilms display similar characteristics to the bulk water bacterial communities, while mature biofilms displayed lower growth rates and lower community richness, indicating a different bacterial community (Boe-Hansen et al., 2002b; Martiny et al., 2003).

#### Characteristics of Drinking Water Sediments

Sediment formation is the result of particle deposition under favorable hydraulic conditions (Vreeburg et al., 2008) (**Figure 3**), and distribution networks can contains as much as 3000 mg/m loose deposits (Barbeau et al., 2005; Vreeburg et al., 2008). Deposited particles offer a favorable environment for bacterial growth, as (i) they provide a large surface area, (ii) are usually composed of organic compounds and also (iii) contain inorganic substrates (e.g., Ca, Fe, Mn; Gauthier et al., 1999; Zacheus et al., 2001). Liu et al. (2014) found that sediments may favor the growth of specific bacterial species, for example bacteria involved in iron and arsenic cycling (e.g., *Rhodoferax* sp. and *Geobacter* sp.). Particle sedimentation combined with biofilm formation and EPS production consolidates the sediment structure, which expands as long as sediments are not re-suspended during high hydraulic peaks (cf. details in section “Biological Stability in Drinking Water: Implications for Treatment and Distribution”). During sediment expansion, anoxic or anaerobic conditions are likely to be created, providing a selective environment for the growth of bacteria not found in the bulk water phase (e.g., *Rhodoferax* sp. and *Geobacter* sp., or bacteria from actinomycetes group; Zacheus et al., 2001; Liu et al., 2014). Sediments were shown to contain large amounts of biomass, in the range of 700 to 4000 ng ATP/g loose deposit (Liu et al., 2014) and up to 10^11^ cells/g (Barbeau et al., 2005) and to harbor the largest bacterial diversity, compared to bulk water and biofilm phases, with 29% of the total bacteria which were not shared with the bulk water (Liu et al., 2014). Sediments can be the source of hygienic and operational problems, as they offer a protective environment for bacteria to grow, particularly for undesirable organisms (Gauthier et al., 1999), and iron-oxidizing bacteria shown to increase corrosion processes (Sun et al., 2014). Furthermore, sediments can be the source of colored water when re-suspended into the bulk water (Vreeburg and Boxall, 2007) and host invertebrates (Christensen et al., 2011).

#### Interactions Between Biofilm, Sediment, and Bulk Water Phases

Mechanisms of interactions between biofilm, sediment, and bulk water bacteria have been investigated to estimate to which extent biofilms and sediments affect the bacterial community in bulk water in terms of abundance and community composition and structure, thus how these interactions might affect biological stability. Biofilms have long been considered as containing the largest fraction (up to 95%) of bacterial cells in drinking water distribution systems (Flemming et al., 2002). However, the sediment phase has been largely overlooked due to sampling difficulties (Liu et al., 2013a). A recent study has shown that 98% of bacterial cells were situated in both biofilms and sediments, of which 60 to 90% were actually situated in the sediment phase (Liu et al., 2014). It was first assumed that the majority of bulk water bacteria originate from biofilm detachment (LeChevallier et al., 1987; van der Wende et al., 1989), rather than bacterial growth in the bulk water phase. However, this hypothesis was challenged by a study by Boe-Hansen et al. (2002a), which demonstrated higher bacterial activity and growth rates in bulk water than in biofilm (0.30 day^-1^ in bulk water compared to 0.048 day^-1^ in biofilm). The study showed that bacterial production in the bulk water constituted 37% of the total bacterial production in a model drinking water system. Recently, Liu et al. (2014) detected different bacterial community compositions in bulk water and biofilm, and Henne et al. (2012) found significantly different core bacterial communities in both water and biofilm sampled from full-scale and long used distribution systems. From these observations, it was suggested that bulk water bacteria function as a seed bank for biofilms and sediments (Henne et al., 2012; Liu et al., 2014), each phase thereafter developing its own bacterial community, with different predominant species depending on specific environmental conditions. However, interactions between bacteria in water, sediment, and biofilm phases are still unclear. Detachment of biofilms and re-suspension of sediments would arguably contribute to bacterial cell concentrations and community composition in the bulk water (cf. details on hydraulic conditions in section “Biological Stability in Drinking Water: Implications for Treatment and Distribution”). Moreover, bacterial cells in biofilm and sediments would compete with bulk water bacteria for available nutrients. As the largest proportion of bacteria is present in the two phases, substantially fewer nutrients would remain available for bulk water bacteria. Consequently, the role and interactions between bulk water, biofilms, and sediments should be taken into account when considering biological stability in drinking water distribution systems.

## Biological Stability in Drinking Water: Implications for Treatment and Distribution

**FIGURE 4:**
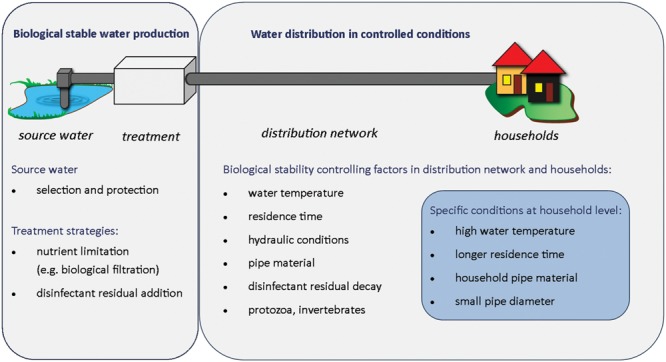
**Biological stability components: source to tap overview of critical parameters controlling biological stability in drinking water systems**.

Both water treatment and distribution conditions can significantly affect biological stability in drinking water distribution systems, by shaping bacterial community characteristics and/or modifying bacterial growth environment. Achieving biological stability therefore requires to (i) produce biological stable water, i.e., a water that does not support bacterial growth, considering the composition of its nutrients and its bacterial community, and (ii) distribute water in conditions that do not promote changes in the microbial community, in full-scale systems as well as in households (**Figure 4**).

### Treatment Strategies for the Production of Biological Stable Water

#### Water Sources and Treatment Strategies

Water treatment strategies are adapted to the characteristics of raw water, which can be very diverse. Deep ground water typically contains very low bacterial cell concentrations (10^3^–10^4^ cells/mL), are often anaerobic and contain low organic nutrients (e.g., AOC below 10 μg ac-C/L) but potentially high methane (e.g., 0.01 to 9 mg/L) and ammonium concentrations (e.g., 0.2 to 5 mg/L; van der Kooij et al., 1982; de Vet et al., 2009; Hedegaard and Albrechtsen, 2014). Surface waters, on the other hand, typically contain high bacterial cell numbers (10^5^–10^6^ cells/mL) and relatively high concentrations of organics (e.g., AOC in the range of 5–150 μg Ac-C/L; van der Kooij, 1990; Hammes et al., 2010a; van der Wielen and van der Kooij, 2010), while numerous in-between situations can be found with, e.g., infiltrated water or phreatic aerobic ground water (van der Kooij et al., 1982; van der Kooij and Hijnen, 1985b). Treatment strategies typically aim to inactivate hygienically relevant organisms, remove micro-pollutants, improve aesthetic aspects (turbidity, taste, and odor), and prevent bacterial growth during water distribution. Therefore, combinations of different treatment types are applied. In several European countries, stable water is produced from surface water using extensive multistep treatments, without the use of residual disinfectant. As an example, treatment trains applied in Zurich and Amsterdam include one or several disinfection steps (e.g., ozonation) and a combination of biological filtration processes (e.g., rapid sand filter, slow sand filter, activated carbon filter, and/or dune infiltration; Hammes et al., 2010a; Vital et al., 2012a). The choice of treatment strategies and combinations are crucial in the production of biological stable water, as it determines (i) composition and concentration of individual organic and inorganic nutrients, and (ii) bacterial community characteristics (abundance, activity, community composition).

#### Oxidation Processes

Oxidation processes, such as chlorination and ozonation or UV/H_2_O_2_ advanced oxidation, are commonly applied as primary disinfection strategies for bacterial inactivation (Bernarde et al., 1967; Hoefel et al., 2005; Ramseier et al., 2011a). Oxidative treatments often result in modification of the substrate composition (van der Kooij et al., 1989; Schmidt et al., 1998; Okuda et al., 2009; Sarathy and Mohseni, 2009; Ramseier et al., 2011b). As an example, Vital et al. (2012a) reported an AOC increase from 20 to 120 μg ac-C/L after ozonation, while DOC concentration did not change, indicating a clear change in the composition of organic compounds in water. In this regard, Hammes et al. (2006) showed that 60–90% of the AOC formed after ozonation of Lake water was composed of organic acids. Primary disinfection processes with high ozone or free chlorine dosage also typically result in inactivation of the entire bacterial community (Hammes et al., 2008, 2010a), thus leaving room for new microorganisms to colonize the treated water and consume the altered nutrient pool. Overall, oxidative processes create highly unstable water due to combined effects of (i) increased nutrients availability and (ii) absence and/or inactivation of bacterial cells, thus creating a new niche for bacterial growth.

#### Biological Filtration Processes

Biological filtration processes are applied worldwide and are usually implemented after primary disinfection processes (van der Kooij, 1992; Prévost et al., 1998; Gauthier et al., 1999; Hammes et al., 2010a; Pinto et al., 2012). Biological filtration processes are believed to be an essential step for production of biological stable water (Rittmann and Snoeyink, 1984; van der Kooij, 1990; Smeets et al., 2009). Different types of filtration technologies, applied for diverse purposes, can act as biological filtration. These include active carbon, rapid or slow sand filtrations, or soil infiltration. When water flows through biological filters, bacterial cells attach to filter particles (Servais et al., 1994; Velten et al., 2007) and consume substrates provided by the water, resulting in formation of a diverse bacterial community. Pinto et al. (2012) recorded the presence of 14 different bacterial phyla in the eﬄuent of a rapid dual media filter. Bacterial community abundance, activity and composition in filters depends on nutrient composition of water flowing through the filter, resulting in the presence of different organisms being able to metabolize different types of substrate, and on environmental parameters, such as temperature (cf. section “A Deeper Look into Microbial Dynamics in Drinking Water”; Fonseca et al., 2001). Bacterial cells regularly detach from the filters, and bacteria are found in water after biological filtration typically in bacterial cell concentrations ranging between 10^4^ and 10^5^ cells/mL (Servais et al., 1994; Hammes et al., 2010a; Lautenschlager et al., 2014). Studies have shown that biological filtration processes shape the bacterial community composition in treated water (Pinto et al., 2012; Lin et al., 2014), which is only slightly modified during water distribution (Henne et al., 2012). Successive biological filtration steps of different types are often applied in order to consume fast degradable and more complex organic compounds. As an example, Lautenschlager et al. (2014) showed in a multi-step treatment plant in Zürich that low molecular weight humic substances were mainly removed during rapid sand filtration, while polysaccharides were degraded in subsequent slow sand filtration. This can be attributed to residence time of water in the slow sand filter, which can be up to 50 times longer than in rapid sand filter (Huisman and Wood, 1974). Application of successive biological filtration steps thus gradually shapes both the nutrient pool and bacterial community characteristics in treated water (Lautenschlager et al., 2014). Changing raw water quality or temperature variations potentially affect bacterial community characteristics in treated water in time, including bacterial community composition (Pinto et al., 2014) and/or activity and/or abundance (van der Wielen and van der Kooij, 2010). Application of biological filtration processes has two main advantages: (i) growth-supporting organic and inorganic nutrients in treated water are considerably reduced (up to 80–90% AOC removal; van der Kooij, 1987; Hijnen and van der Kooij, 1993; Servais et al., 1994) and (ii) a very diverse autochthonous bacterial community is released in treated water, therefore covering a large substrate utilization spectrum and contributing to biological stability of water (cf. section “A Deeper Look into Microbial Dynamics in Drinking Water”).

#### Membrane Filtration

In recent years, application of membrane filtration methods rather than chemical additions to water, have been proposed as an alternative primary or secondary disinfection approach (Sibille et al., 1997; Sibille, 1998; Vrouwenvelder et al., 2004). Several membrane types can be distinguished based on their removal capacity, and include MF, UF, NF, and RO (Mallevialle et al., 1996). The advantage of this approach is that it lowers significantly bacterial cell concentration in treated water, by removing over 99.5% of bacterial cells (Liikanen et al., 2003), without producing any by-products that have potential adverse health effects, or contribute to available nutrients for bacterial growth. Moreover, membrane filtration considerably reduces the amount of suspended particles entering the distribution system, thus lowering the potential for sediment deposition (Vreeburg et al., 2008) and associated problems (cf. sections “Location of Bacterial Cells: In Water, Sediment, and Biofilm” and “Water Temperature”).

The use of membrane-based filtration methods, however, potentially leads to production of unstable water (Okabe et al., 2002). While bacterial cells are physically removed from the water, a significant fraction of nutrients passes through the membranes, whatever the cut-off value (Liu et al., 2013b). While over 90% of BDOC is retained with NF membranes (Escobar and Randall, 2001; Vrouwenvelder et al., 2004), a large proportion of AOC remain in the filtered water, ranging between 5 and 90%, depending on the cut-off value and the influent water quality (Liikanen et al., 2003; Meylan et al., 2007). Even RO membranes, which enable an efficient bacterial cell and organic compound removal (over 99.5% rejection of bacterial cells, and over 90% of TOC rejection), do not retain all AOC, with about 80% AOC rejection efficiency (Park and Hu, 2010). As no system remains sterile (Liikanen et al., 2003; Liu et al., 2013b), these nutrients are fully available for new bacterial growth in the distribution system. In such case, water retains a certain level of growth potential, as highlighted by Liikanen et al. (2003) who observed an HPC increase of over a log unit during incubation of the permeate of nanofilters for 20 days. Similarly, significant regrowth occurred in a model distribution system fed with RO permeate, both in the bulk water (from 50 bacterial cells/mL in the RO permeate up to about 10^3^ cells/mL in the bulk water after 20 days residence time)and in the form of a biofilm (containing 8 × 10^3^ cells/cm^2^) developed on PVC coupons in a biofilm reactor fed with the RO-treated water during 20 days. Bacterial cell numbers remained, however, extremely low in comparison with a similar system fed with the same tap water without prior RO treatment (2 × 10^5^ cells/mL in bulk water, and 7 × 10^5^ cells/cm^2^ in biofilm), showing a reduced growth potential of the water after RO filtration. In summary, membrane filtered water remains unstable due to the permeability of certain compounds through the membrane, the lowest growth potential being obtained by membranes retaining the highest proportion of nutrients (e.g., RO).

#### Ion Exchange

Ion exchange has also been proposed as an additional step in full-scale conventional drinking water treatment system to improve color of final water and biological stability (e.g., Heijman et al., 1999). IEX is efficient in removing low molecular weight organics (Bolto et al., 2002), and has been shown to significantly reduce both DOC (e.g., by 50%) and AOC (e.g., by 60%) of final water (Grefte et al., 2011). Biofilm formation rate of produced water was subsequently reduced by 70%. However, water treated by IEX retains a certain degree of bacterial growth potential: in a study by Liu et al. (2013b), bacterial growth was detected in a water treated with IEX and incubated for 24 h, with an increase in ATP of 23% and in total bacterial cell concentration of 41%, showing that the IEX treatment did not fully prevent bacterial growth.

#### Secondary Disinfection

In many cases, a secondary disinfection is applied to the produced drinking water before distribution, typically with free chlorine, chlorine dioxide, or monochloramine (van der Kooij, 1992; LeChevallier et al., 1996; Prévost et al., 1998; Batté et al., 2006; Pinto et al., 2012). The aim of secondary disinfection is either to prevent bacterial growth in drinking water distribution systems, or to reduce HPC values below the guideline value. In both cases, application of a disinfectant alters significantly the bacterial community composition and structure, usually resulting in a lower diversity and richness, as well as the nutrient pool (cf. details in section “A Deeper Look into Microbial Dynamics in Drinking Water”). Consequently, the growth potential of the water is affected with new niches available for bacterial growth once disinfectant residual is depleted (cf. section “A Deeper Look into Microbial Dynamics in Drinking Water”).

### Factors Influencing Biological Stability in Distribution Networks

Water distribution conditions can have a considerable impact on biological stability (**Figure 4**). Various factors influence microbial processes described in Section “A Deeper Look into Microbial Dynamics in Drinking Water,” including water temperature, residence time, hydraulic conditions, pipe material, and/or disinfectant residual decay. An overview of microbial dynamics in a drinking water pipeline section is provided in **Figure 3**.

#### Pipe Materials

Pipe material composition influences biofilm development on pipe surfaces (**Figure 3**). A single distribution system typically includes diverse materials such as metal pipes (e.g., cast iron, stainless steel), cement, and/or synthetic polymers (e.g., PVC), on which widely different growth rates, bacterial densities, and community compositions were measured (Niquette et al., 2000; Yu et al., 2010; Wang et al., 2014). Several studies have demonstrated that iron pipes allow the highest bacterial densities, with up to 45 times higher bacterial biomass fixed on iron coupons than on plastic materials (Norton and LeChevallier, 2000; Niquette et al., 2000). Corrosion of iron pipes leads to the release of particles and deposit formation (**Figure 3**), on which organic and inorganic compounds adsorb, and which act as attachment sites, where bacteria are protected from disinfectant residuals (Camper, 2004; Morton et al., 2005). Synthetic polymeric pipe materials, such as cross-linked poly-ethylene (PEX), polybutylene (PB), or PVC, were shown to release biodegradable organic substances (**Figure 3**), modifying the available nutrient source for bacteria to grow (Skjevrak et al., 2003; Bucheli-Witschel et al., 2012). Even if stable water is produced at the treatment plant, release of additional nutrients into water can cause biological instability. As an example, van der Kooij and Veenendaal (2001) reported an increase by up to 200% in ATP concentration after incubation of water with plastic materials (e.g., plasticized PVC, PVCp) compared to the incubation of the same water alone. Pipe materials influence the bacterial community predominantly during its first stage of development (Martiny et al., 2003; Henne et al., 2012). Consequently, construction of new distribution systems or replacement of pipe segments in old distribution systems profoundly affects the biological stability of drinking water for a period of time that can reach up to several years before a stable system is reached again (Martiny et al., 2003).

#### Hydraulic Conditions

Hydraulic changes in distribution systems are frequent and play a major role in interactions between bulk water, sediment, and biofilm phases (**Figure 3**). Low water consumption periods result in low flow velocities or even water stagnation in reservoirs and parts of distribution systems, enabling particle deposition, increased residence time, and offering favorable conditions for bacterial growth to occur (Gauthier et al., 1999; Zacheus et al., 2001; Liu et al., 2013a,b). On the other hand, hydraulic peaks caused by high consumption periods, fire-fighting actions, pipe flushing or system malfunctioning such as pipe breaks, unavoidably result in increased biofilm detachment and possible sediment re-suspension (Lehtola et al., 2006; Vreeburg and Boxall, 2007). This increases bacterial dispersal in the network water and will modify bacterial abundance and community composition in the bulk water, thus affecting biological stability (**Figure 3**). Hydraulic peaks can cause release of hygienically relevant organisms (Torvinen et al., 2004; Wingender and Flemming, 2011), and serious aesthetic issues such as presence of invertebrates in the bulk water (van Lieverloo et al., 2002a), or color deterioration of water at the point of consumption (Vreeburg et al., 2004). To avoid such problems, Vreeburg et al. (2009) have proposed to adapt distribution system design, to control hydrodynamic conditions in distribution systems and limit particle deposition, sediment formation, and avoid dramatic hydraulic peaks.

#### Water Temperature

Water temperature is an essential factor influencing bacterial growth kinetics and competition processes. Drinking water temperatures typically range between 3 and 25°C in European countries, (Kerneis et al., 1995; Niquette et al., 2001; Uhl and Schaule, 2004), and fluctuate seasonally within this temperature range even within a single drinking water distribution system. Elevated water temperatures have often been associated with increased bacterial abundance in drinking water distribution systems (Servais et al., 1992; Kerneis et al., 1995; Francisque et al., 2009; Liu et al., 2013a), and with higher numbers in indicator organisms such as coliforms or *Aeromonas* (Burke et al., 1984; Volk and Joret, 1994; LeChevallier et al., 1996). Francisque et al. (2009) recorded five times more occurrences of HPC concentrations above 100 CFU/mL at temperatures above 18°C than at lower water temperatures. In addition, water temperature can also affect bacterial community composition, by providing competitive advantages to specific bacterial species in defined temperature ranges, including pathogenic species (Vital et al., 2007, 2012b). For example, Vital et al. (2012b) showed that the maximum growth rate and competitive fitness of *E. coli* grown with an indigenous drinking water community increased with temperature in the range of 12–30°C. There is therefore increased chance for problems associated with bacterial growth in summer periods (with higher water temperatures), such as hygienic risks, deterioration of aesthetic aspects of water, malfunctioning of water installations, exceeding of legal guidelines, for e.g., heterotrophic plate counts (cf. section “Problems Associated with Bacterial Growth in Drinking Water Distribution Systems”). In this regard, specific attention is given to the influence of anticipated global warming on drinking water quality, as average water temperatures are expected to increase, concomitantly with longer periods at higher temperatures (Levin et al., 2002). Moreover, significant seasonal shifts in bacterial community composition have been reported in eﬄuents of treatment utilities (Pinto et al., 2014). Though the cause of such variations is not clear at this stage, seasonal variations in water temperature could well be involved in bacterial community characteristics of water entering the distribution system. Further research would be needed to determine to which extent these changes affect bacterial competition processes within the drinking water distribution system.

#### Residence Time

Residence times can reach up to a few days within a distribution system (Kerneis et al., 1995), leaving time for bacterial growth to occur. Residence times depend on (i) distance from treatment plant, up to 100 km or more in the case of extended cities or remote villages (Cook et al., 2014), (ii) pipe diameters, varying from a few meters in water mains down to a few millimeters in service pipes, and (iii) water flow velocity caused by water consumption. The latter also influences additional residence time of water within reservoirs at the treatment outlet and/or within distribution systems. In general, higher bacterial abundances were observed at higher water residence times in the network (Maul et al., 1985; Kerneis et al., 1995; Uhl and Schaule, 2004; Nescerecka et al., 2014). In the case of chlorinated water, increased bacterial abundance at long residence time is often congruent with decay of disinfectant residual (Servais et al., 1995). However, in systems distributing water without residual disinfectant, long residence times do not systematically lead to an increase, but occasionally to a decrease in bacterial abundances and/or activity (van der Wielen and van der Kooij, 2010), possibly be due to substrate limitations. In summary, the effect of residence time on microbial dynamics is not clear and is often dependent on other factors such as residual disinfectant decay and/or substrate availability.

#### Residual Disinfectant Decay

While biological stability could theoretically be achieved by maintenance of sufficient disinfectant residuals at all points of a distribution system, this is challenging to achieve. Many of the factors mentioned in this section contribute to disinfectant decay within distribution systems. For example, dissolved nutrients in bulk water, as well as EPS and organic and inorganic nutrients adsorbed on biofilms and sediments can react with the disinfectant (Gauthier et al., 1999; Campos and Harmant, 2002), resulting in lowered concentrations or even absence of residual disinfectant at long residence times (Maul et al., 1985; Kerneis et al., 1995). This phenomenon is increased in pipe sections with small diameter, in which the surface to volume ratio is higher, thus increasing contact of water with pipe materials and/or biofilms and sediments. Prévost et al. (1998) have observed a faster decay in 150 mm diameter pipes than in larger water mains. Chemical reactions are further affected by water temperature, also modifying the disinfection capacity in time (Bernarde et al., 1967; Urano et al., 1983). Loss of disinfection residuals undoubtedly results in biological instability and bacterial regrowth (Servais et al., 1992; LeChevallier et al., 1996; Nescerecka et al., 2014).

#### Construction, Operation, and Maintenance Practices

Good practices for construction, operation, and maintenance of water distribution systems are also essential to maintain biological stability in distribution systems (Smeets et al., 2009). A code for hygiene during pipe installation was developed by water utilities in the Netherlands (van Lieverloo et al., 2002b). Besides, maintenance of sufficient pressure in the system for protection against intrusion of external water, and good maintenance of pipelines to maintain physical integrity and limit leakages, are essential to prevent external contamination. In this way, intrusion of both external organisms and organic and inorganic nutrients is avoided, that would profoundly affect the nutrient pool and bacterial community composition in drinking water.

### Is Biological Stability Compromised at Household Level?

Premise plumbing conditions can cause biological instability and compromise water quality in the last meters prior to consumption. Water stagnation in household pipes has been shown to result in significantly increased bacterial abundance in the water (up to threefold increase) and in a shift in bacterial community composition (changes ranging from 20 to 100% compared to the population in the flushed water; Pepper et al., 2004; Lautenschlager et al., 2010; Lipphaus et al., 2014). Water quality within households is influenced by the same parameters as in the distribution system, i.e., water temperature, residence time, pipe material, hydraulics, disinfectant decay, and interactions between bulk water, sediments, and biofilms. However, conditions within households are more extreme than the water in the distribution network (**Figure 4**). Average water temperatures are generally higher in households than in the distribution system, sometimes reaching 20°C or more, due to pipes installed through heated rooms or nearby heat sources (Lautenschlager et al., 2010; Lipphaus et al., 2014). Bacterial growth is supported in biofilms and bulk water due to both warmer water temperatures and long residence times in household pipes. On average, water is stagnant in household pipes for 23 h per day (Proctor and Hammes, 2015), and in practice this varies from a few hours up to some weeks in cases such as holiday houses or seasonal hotels. Measured changes in microbial community abundance and composition were dependent on stagnation time (Lautenschlager et al., 2010; Manuel et al., 2010). One of the challenges in maintaining good water quality up to the consumer’s tap is that pipe materials choice and replacement are left to the house owner responsibility, and are often not well controlled. A large variety of pipe materials are often found in households and differ from the ones found in distribution mains. These include copper, plastic, and elastomeric materials, which are sometimes not in accordance with regulations for use in drinking water (Regulation EC No 1935/2004, 2004; Regulation EU No 10/2011, 2011). Typical examples are flexible plastic materials, such as shower tubes or small rubber fittings, including ethylene-propylene-diene-monomer (EPDM), which have considerable bacterial growth promotion potential (Bucheli-Witschel et al., 2012). The effect of pipe material on bacterial growth is further increased by significantly smaller pipe diameters in households than in distribution networks, resulting in increased contact between bulk water bacteria and biofilm and/or pipe material, and in faster disinfectant decay (Servais et al., 1992; Rossman et al., 1994; Prévost et al., 1998). Finally, consumer’s taps can be the source of water back-contamination by organic and inorganic nutrients and/or bacteria. Better regulations of pipe materials in use in premise plumbing would help a better control of microbial processes in the last meters before the tap (Flemming et al., 2014; Proctor and Hammes, 2015). However, it is still unclear if household conditions could promote uncontrolled bacterial growth and significant biological instability, following a biological stable drinking water distribution system.

## How is Biological Stability Assessed?

There are three types of indicators for biological instability of a drinking water system. Firstly, indirect signs of instability for water utilities are customer complaints about taste, color, turbidity, and/or odor, and the deterioration or malfunctioning of water installations, due to fouling or (bio-) corrosion. Secondly, biological stability/instability is traditionally predicted based on growth-promoting properties of treated water and/or materials in contact with water, and associated with guideline values (Supplementary Table S1). Thirdly, direct detection of changes in microbial community characteristics within distribution systems is indicative for instability. This section reviews existing and emerging methods for predicting and monitoring biological stability in drinking water distribution systems (**Figure 2**).

### Predictive Methods

#### Evaluation of Growth Promoting Properties of Drinking Water and Associated Guidelines

A range of methods have been developed to assess growth promoting properties (growth potential) of drinking water and are traditional indicators for biological stability (van der Kooij et al., 1982; Servais et al., 1989; Hu et al., 1999; Ross et al., 2013). In essence, these methods are predictive, as the water is analyzed before distribution, and the tests are used to predict the extent of growth that could potentially occur during water distribution.

Initial focus of these methods was on biodegradable organic carbon and included the assessment of AOC, initially proposed by van der Kooij et al. (1982), van der Kooij and Hijnen (1985b), and BDOC method, proposed by Servais et al. (1987, 1989). Both methods have been the subject to numerous adaptations for improving the tests representativeness, ease of handling and time (Werner, 1984; Joret and Levi, 1986; Joret et al., 1988; Lucena et al., 1990; Ribas et al., 1991; Hambsch et al., 1992; LeChevallier et al., 1993; Miettinen et al., 1997; Haddix et al., 2004; Hammes and Egli, 2005; Sack et al., 2009; Ross et al., 2013). While the AOC assays by definition focus on easily available substrates for planktonic growth, BDOC assays enable the assessment of the refractory fraction of biodegradable organic carbon, which can be used by biofilm-bacteria in distribution systems (Camper, 2004; Flemming and Wingender, 2010). In general, higher BDOC values than AOC values are measured in drinking water. For example, Volk and LeChevallier (1999) reported BDOC values in the range of 0.15–0.75 mg/L and AOC values in the range of 0.10–0.33 mg/L in treated surface waters. Systematic application of the methods for full-scale studies showed that little AOC uptake occurred and HPC values remained below guideline values (100 cfu/mL) during distribution of non-chlorinated waters with an AOC level below 10 μg Ac-C/L (van der Kooij, 1992). In chlorinated water, statistically fewer occurrences of coliforms were observed at AOC concentrations below 100 μg Ac-C/L than in waters with higher AOC concentrations (LeChevallier et al., 1996). No decrease in BDOC was observed during distribution of waters with a BDOC levels below 150 μg C/L in chlorinated distribution systems (Servais et al., 1992; Volk and Joret, 1994). These different AOC and BDOC values have been associated with no/limited bacterial growth and have been subsequently used as guidelines for biological stable water (van der Kooij, 1987; LeChevallier et al., 1992).

Both AOC and BDOC methods focus primarily on organic carbon as the only growth-limiting substrate in drinking water. However, other compounds were identified as possible microbial growth controlling substances (organic carbon, ammonium, manganese, iron; Rittmann and Snoeyink, 1984; States et al., 1985; van der Kooij and Hijnen, 1985b; Miettinen et al., 1997), and might be in some cases more critical to describe and understand microbial dynamics in full-scale drinking water systems. The bacterial growth that water can support as such, independently of its growth-limiting element, can be assessed by incubation of a water samples without pre-treatment in controlled conditions (Gillespie et al., 2014). The same approach can be extended for the assessment of growth-limiting compounds in water samples by step-wise addition of single substrates or combinations of substrates (Miettinen et al., 1999; Ihssen and Egli, 2004), and subsequent measurement of the bacterial growth potential.

A large number of devices have been used to study the property of water to support development of biofilms in drinking water distribution systems. These include annular reactors (e.g., Volk and LeChevallier, 1999), Propella^®^ reactors (e.g., Lehtola et al., 2006), flow cell systems (e.g., Manuel et al., 2010), and Robbins devices (e.g., Kalmbach et al., 1997). An extensive overview of available systems has been provided by Gomes et al. (2014). Many of these devices comprise coupons of defined materials such as copper, PVC or cement, which can have an influence on growth of biofilm. Alternatively, van der Kooij et al. (1995) proposed to quantify the potential of water to form biofilms on inert materials. Subsequently, additional guideline values for biological stable water have been introduced, such as BFR which should be kept smaller than 10 pg ATP/cm^2^.d in produced drinking water (van der Kooij et al., 1995).

#### Growth Promoting Properties of Materials in Contact with Water

Materials in contact with drinking water can profoundly influence growth potential. A number of assays have been developed to assess this effect, and the approach varies from country to country. For example, a standardized method in the United Kingdom measures consumed dissolved oxygen as an indicator for bacterial growth on 150 cm^2^ of material in contact with water during 7 weeks (Colbourne and Brown, 1979; BSI, 2000). In Germany, formation of biomass on 800 cm^2^ material surface is measured as the volume of slime produced during 12 weeks (ÖNorm B 5018-12, 2002) and in the Netherlands, BFP is measured on 50 cm^2^ material (van der Kooij and Veenendaal, 1994) with ATP analysis. The latter test was further adapted to estimate the potential of tested material to promote bacterial growth both on its surface (biofilm) and in the bulk water in contact with the material (BPP; van der Kooij and Veenendaal, 2001). More recently, Bucheli-Witschel et al. (2012) have developed a new test package which requires 14 days and that measures the BPP (quantified with FCM) as well as the AOC fraction in water after a series of high-temperature standardized migration assays. Though assessment of growth-promoting properties of materials in contact with water is a useful decision-making tool for selection of appropriate materials, it is challenging to assess growth-promoting properties of materials already installed in networks. The latter can be largely affected by long-term aging in specific conditions in distribution systems, including continuous flow and presence of biofilms, sediments and/or specific degrading organisms.

### Direct Indicators of Change

Distribution conditions can significantly affect bacterial growth in distribution systems (cf. section “Biological Stability in Drinking Water: Implications for Treatment and Distribution”). To evaluate the distribution effect, the best approach is to directly characterize bacterial communities in the system. A change in bacterial abundance, viability and/or community composition can be considered as indicative for biological instability.

#### Bacterial Abundance

An increase in bacterial abundance is a clear sign of biological instability, and can be measured as a change in specific bacteria, bacterial groups, or in the total bacterial community (Servais et al., 1992; LeChevallier et al., 1996; van der Wielen and van der Kooij, 2010; Nescerecka et al., 2014). Specific detection usually focuses on hygienically relevant organisms such as *Legionella, Mycobacterium, Pseudomonas aeruginosa*, total coliforms, *Enterococcus* and *E. coli* (Tallon et al., 2005). In the Netherlands, the presence of *Aeromonas* is also included in legislation and often tested as an indicator for biological stability (Waterleidingbesluit, 2001). Specific detection is traditionally performed by cultivation on selective media, and more recently with molecular-based techniques such as qPCR or with the use of specific antibodies (Rompré et al., 2002; Schets et al., 2002; Tallon et al., 2005; Hügler et al., 2011; Douterelo et al., 2014). Besides specific detection, cultivation is also used worldwide in drinking water monitoring for HPC methods (Allen et al., 2004; Pepper et al., 2004; Uhl and Schaule, 2004; Batté et al., 2006). There is more or less universal agreement that the fraction of bacterial cells detected by HPC methods is less than 1% of the total bacterial concentration in drinking water (Staley and Konopka, 1985; Hoefel et al., 2003; Hammes et al., 2010a; Epstein, 2013). Quantification of all bacterial cells in a water sample is achieved by cell labeling with fluorescent dyes (e.g., SYBR Green I or DAPI) and subsequent detection by either epifluorescence microscopy (Servais et al., 1992; Niquette et al., 2001) or FCM (Hoefel et al., 2003; Hammes et al., 2008). FCM enables extremely fast water analysis with limited sample handling (Hammes et al., 2008; Prest et al., 2013), as well as possibility for automation (Hammes et al., 2012; Besmer et al., 2014). The use of FCM has revealed an increase in total cell concentrations in the range of 11–23% in drinking water distributed without disinfectant residual (Hammes et al., 2010a; Prest et al., 2014) and up to 300% in household taps after stagnation (Lautenschlager et al., 2010). While all methods above are well adapted for enumeration of suspended bacterial cells, they cannot be applied directly for enumeration of bacteria attached to particles, sediments, or biofilms. Pre-treatments such as ultrasonication or physical removal (e.g., swabbing or scratching the surface) are required to detach and suspend bacteria (Boe-Hansen et al., 2002b; Magic-Knezev and van der Kooij, 2004).

#### Bacterial Viability and Activity

A large number of viable drinking water bacteria is not detected with cultivation methods as a result of being either so-called unculturable bacteria (Kaeberlein et al., 2002; D’Onofrio et al., 2010; Epstein, 2013) or in the so-called “VBNC” state (Oliver, 2005; Hammes et al., 2011). It is particularly useful to determine cultivation-independent bacterial viability/activity when disinfection is applied. Viability of all bacterial cells can be assessed by cell labeling with fluorescent dyes targeting specific features of bacterial cells related to bacterial viability, such as cell membrane integrity (e.g., propidium iodide), membrane potential [e.g., DiBAC_4_(3)], respiratory activity (e.g., CTC), and subsequent detection with epifluorescence microscopy or flow cytometry (Prévost et al., 1998; Hoefel et al., 2005; Berney et al., 2008; Ramseier et al., 2011a; Hammes et al., 2011). As an example, Berney et al. (2008) have shown that water sampled at a household tap after non-chlorinated water distribution contained 1.7 × 10^5^ cells/mL, of which about 70% had intact, polarized bacterial cell membranes, but only 20% displayed esterase activity. Noticeable biological instability has been detected in a chlorinated drinking water distribution system, in which the total bacterial cell concentration increased from 1.62 × 10^5^ to 1.07 × 10^6^ cells/mL and the percentage of cells with intact membranes increased from 3 to 59% (Nescerecka et al., 2014). Besides the above single-cell methods, ATP is a useful bulk water measurement of biological activity, as ATP is present only in living cells (Karl, 1980). Typically, total ATP concentrations in the range of 0.8 to 12 ng ATP/L are found in drinking water (Lautenschlager et al., 2010; van der Wielen and van der Kooij, 2010). For increased specificity in the method, it is possible to distinguish between intracellular and extracellular ATP and to estimate the average amount of bacterial ATP per cell when combined with FCM quantification (Hammes et al., 2010b; Vital et al., 2012a). ATP measurements have successively been applied in several case studies to assess biological stability (van der Wielen and van der Kooij, 2010; Vital et al., 2012a; Liu et al., 2014; Nescerecka et al., 2014). A few alternatives for bacterial activity measurements in drinking water have been proposed (Staley and Konopka, 1985), including the measurement of H^3^-Leucine or H^3^-thymidine incorporation (Servais et al., 1992; Boe-Hansen et al., 2002a).

#### Bacterial Community Composition

A change in microbial community composition is indicative of instability (Lautenschlager et al., 2013; Pinto et al., 2014; El-Chakhtoura et al., 2015). Molecular methods for this purpose are predominantly based on DNA extraction and PCR amplification (e.g., of 16S rRNA gene) and are roughly classified into either fingerprinting methods or high-throughput sequencing methods (Read et al., 2011; Douterelo et al., 2014). Fingerprinting methods such as DGGE and T-RFLP provide insight in the bacterial community composition, without identification of specific bacterial groups or species. Fingerprinting methods are useful for a quick comparison between bacterial community in different water samples, and have been applied to study, e.g., the effect on bacterial communities of different pipe materials (Roeder et al., 2010; Yu et al., 2010) or of water stagnation and flushing of household taps (Lautenschlager et al., 2010; Manuel et al., 2010). Recently, non-molecular techniques such as flow cytometric fingerprints have been shown to be indicative of the bacterial community composition, and have been useful for detection of bacterial community shifts after changing environmental conditions or after water distribution (De Roy et al., 2012; Koch et al., 2013; Prest et al., 2014).

16S rRNA-gene based NGS methods, including 454-pyrosequencing, Illumina, or Ion-torrent, enable taxonomic and/or functional classification of organisms present in drinking water at various phylogenetic levels. These high throughput techniques evolved rapidly in the past decade and are consequently used in increasing numbers of studies on drinking water (e.g., Hwang et al., 2012; Wang et al., 2013b, 2014; Pinto et al., 2014; Lin et al., 2014; Liu et al., 2014; Roeselers et al., 2015). 16S rRNA gene based NGS techniques also enable the study of specific bacterial groups such as ammonia-oxidizing, iron-oxidizing, or sulfate-reducing bacteria (Gomez-Alvarez et al., 2013; Sun et al., 2014).

## Evaluation of Current Definitions and Approaches for Biological Stability Assessment

### Existing Definitions of Biological Stability

The first definition of biological stability was formulated by Rittmann and Snoeyink (1984): “*A biological stable water does not support the growth of microorganisms to a significant extent, whereas an unstable water supports high numbers of microbes in distribution systems if sufficient disinfectant is not used*”. This definition essentially focused on the properties of water leaving treatment facilities. However, concomitantly with research developments on microbial dynamics in drinking water treatment and distribution systems (Supplementary Table S1), it became clear that not only the properties of water, but also distribution conditions could significantly affect bacterial growth in drinking water distribution systems (cf. section “Biological Stability in Drinking Water: Implications for Treatment and Distribution”). New definitions were proposed taking into account combinations of parameters: Sibille (1998) pointed out the importance of organic matter and of predation by protozoa (Supplementary Table S1, definition Nr. 2), while van der Kooij (2000) considered both the properties of treated water and of piping material as critical points: *“Biostability is defined as the inability of water or a material in contact with water to support microbial growth in the absence of a disinfectant”.* With the emergence of methods such as flow cytometry and high-throughput sequencing methods, sensitive detection of changes in bacterial community characteristics led to a broader definition of biological stability (Lautenschlager et al., 2013): “*Biological stability would imply no changes occurring in the concentrations and composition of the microbial community in the water during distribution*”.

The last two definitions strongly rely on available methods, and shaped the two main current strategies for assessment of biological stability, namely (i) prediction and (ii) direct assessment of changes in bacterial community characteristics during water distribution. Both approaches have major advantages and drawbacks, as detailed in this section and summarized in **Table 1**.

**Table 1:** Summary of advantages (+) and drawbacks (–) of current approaches for assessment of biological stability of drinking water.

Predictive approach		Direct assessment approach	
*AOC, BDOC, BFR, BPP guidelines values*		*No change in microbiological-related parameters*	
+	Useful decision-making tools	–	Lack for guideline values and specified methods
–	Guidelines sometimes too strong	–	No clear value for what is an acceptable change
–	Distribution system complexity not considered	+	Both water and system stability are considered
–	No evaluation of what really happens	+	Direct evaluation of what really happens
–	Guideline values dependent on application or not of disinfectant residuals	+	Applicable to any system (w/o disinfectant residuals)
–	Focus on heterotrophic growth	+	All bacterial types considered
–	Focus on bacterial abundance	+	All characteristics of bacterial community considered (abundance, viability, composition)

### Evaluation of Predictive Approaches

One usual approach to evaluate biological stability is the prediction of changes that could potentially occur during water distribution, based on controlled laboratory-scale methods (e.g., AOC, BDOC, BFR, BPP tests; van der Kooij et al., 1982; LeChevallier et al., 1993; van der Kooij and Veenendaal, 2001; Bucheli-Witschel et al., 2012) and/or modeling of microbial dynamics during water distribution (Servais et al., 1992, 1995; Dukan et al., 1996; Srinivasan and Harrington, 2007). The approach is based on the definition of biological stability provided by van der Kooij (2003), who subsequently proposed to use combinations of tools for (i) assessment of biological stability of water by evaluating treated water growth-promoting properties based on both organic carbon content (AOC method) and biofilm promoting properties (BFR method), (ii) assessment of growth-promoting properties of materials in contact with water (e.g., BPP test) and (iii) modeling effects of water quality parameters and distribution systems conditions on microbial activity. In this way, both effects of water growth potential and distribution conditions are evaluated, and the effect of each individual parameter, such as water temperature, residence time, residual disinfectant decay, or hydraulic conditions can be modeled or tested individually (Dukan et al., 1996; van der Kooij, 2003). Thorough application of predictive methods and statistical evaluation of large datasets from distribution systems (van der Kooij, 1992; Servais et al., 1995; van der Kooij et al., 1995; LeChevallier et al., 1996) have provided guideline values (e.g., for AOC, BDOC, or BFR or BPP parameters) that are helpful decision-making tools for water utilities for optimization of water treatment trains for production of biological stable water and of distribution (cf. Supplementary Table S1 and section “How is Biological Stability Assessed?”).

However, predictive approaches do not cover all aspects of bacterial growth-controlling factors in drinking water distribution systems. As an example, AOC tests by definition do not evaluate autotrophic growth or limitations in any other nutrient that organic carbon, unless the method is adapted and specifically targets inorganic nutrient limitation. Besides, conditions in full-scale distribution systems are complex, with the conjunction of numerous factors that are specific to each and every distribution system: structure and length of distribution systems, water consumption and temperature profiles, combination of pipe materials, history of water source and treatment implementations, pipe replacement, and/or maintenance actions such as pipe flushing. These factors would influence the bacterial community that colonize drinking water distribution systems (cf. details in sections “A Deeper Look into Microbial Dynamics in Drinking Water” and “Biological Stability in Drinking Water: Implications for Treatment and Distribution”). As a result, prediction does not necessarily reflect what actually occurs during water distribution system, as many factors are likely to affect bacterial dynamics.

### Evaluation of Direct Assessment Approaches

Another approach to assess biological stability relies on measurement of changes in bacterial community characteristics directly in distribution systems (Hammes et al., 2010a; Lautenschlager et al., 2013), and is based on the definition of biological stability provided by Lautenschlager et al. (2013). This approach has the major advantage to directly evaluate what occurs in drinking water distribution systems and/or at household levels, and to take into account both effects of treated water and of distribution conditions. Besides, the approach is applicable to any drinking water distribution system, whether disinfectant residuals are present or not, and all bacterial types are considered, including heterotrophic and autotrophic bacteria. Finally, absence of change in bacterial community characteristics refers to any parameter related to microorganisms in water, i.e., not only abundance, but also viability, activity and composition and structure of the microbial community.

However, the absence of a defined toolbox and clear guideline values represents a major difficulty for water utilities to implement the direct approach. There is currently no specification of which parameters should be measured, as there is no unified method to describe and quantify microorganisms and particularly bacterial communities in water. Most likely different conclusions could be obtained from different methods, as they target part or all the bacterial community, and/or specific features related to bacterial activity or viability (cf. details in section “How is Biological Stability Assessed?”), and direct comparison of results from different studies is difficult. More importantly, there is a major knowledge gap, related to the “degree of acceptable change” or the degree of instability that would cause problems such as deterioration of water aesthetic aspects or of installations. During studies performed with flow cytometry, small changes in bacterial cell abundance have been detected in drinking water distribution systems (from 9.5 ± 0.6 × 10^4^ to 1.3 ± 0.1 × 10^5^ cells/mL; Lautenschlager et al., 2013; from 3.5 ± 0.2 × 10^5^ to 4.3 ± 0.4 × 10^5^ cells/mL; Prest et al., 2014). While Hwang et al. (2012) and Pinto et al. (2014) have shown that the core microbial community does not change with distance in the water distribution system, El-Chakhtoura et al. (2015) highlighted an extreme dynamicity in rare taxa (3–4% of the total drinking water bacterial community). These relatively small variations in bacterial community characteristics were not related to any loss in water quality, and it is unclear from these studies if such a change in bacterial abundance and/or community composition should be considered as a problem, or more broadly as biological instability. One essential question is whether universal guideline values can be proposed, for e.g., bacterial cell concentrations and/or extent of acceptable change, or whether the degree of acceptable change is specific to each and every drinking water distribution system.

## View: A Multi-Disciplinary Approach for the Assessment of Biological Stability

### New Definition of Biological Stability

In essence, a biological stable drinking water is a water where the microbial community does not change during distribution, or at least not to a degree that affects negatively consumer’s safety and aesthetic perception or cause technical system failure, neither on spatial nor on temporal scales, at any drinking water distribution location including the point of use and consumption. From a principle definition perspective, biological stability would imply that no change in the microbial community characteristics (abundance, viability, community composition) is detected in time or distance in the distribution system. Practically, current knowledge still does not allow for stating specific guideline values of acceptable change in the water quality.

**FIGURE 5:**
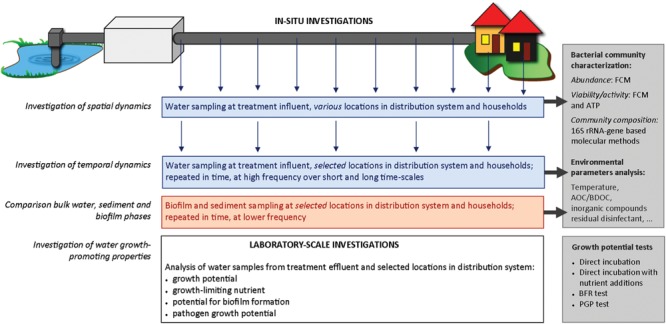
**Suggested approach and methods for studying biological stability in drinking water distribution systems**.

Achieving biological stability requires that (i) biological stable water is produced and (ii) distributed in conditions that do not promote uncontrolled changes in the microbial community, until the point of consumption (**Figure 4**). Consequently, we propose a comprehensive, integrated approach for the study of bacterial dynamics in drinking water distribution systems, requiring the use of both predictive methods in controlled laboratory tests for the assessment of water growth-promoting properties, and analytical methods for direct on-site spatial and temporal monitoring of bacterial communities (**Figure 5**). Parts of the comprehensive approach can be selected depending on the application, for e.g., regular water monitoring, collection of information toward targeted improvement of treatment or distribution conditions, or in-depth research (cf. details in section “Applications”).

### *In Situ* Assessment of Spatial and Temporal Bacterial Dynamics in Distribution Systems

Designing an *in situ* sampling strategy is a crucial step for assessing biological stability. The sampling strategy should cover different aspects of water microbial quality variations in drinking water distribution systems:

• Assess spatial variations in bulk water quality in distance during water distribution, due to either intrinsic bacterial growth potential of the water and/or influences of distribution conditions (section “Biological Stability in Drinking Water: Implications for Treatment and Distribution”). The sampling scheme should therefore include the treatment eﬄuent and various locations in the distribution system. Nescerecka et al. (2014) described a randomized sampling approach for so-called hot-spot detection. As alternative, directed sampling can be considered based on hydraulic models/calculations, tracer tests, and/or pressure zones, to select distribution areas of interest (e.g., based on residence time in the system or distance to the treatment plant; van der Wielen and van der Kooij, 2010; Lautenschlager et al., 2013; Liu et al., 2014).
• Assess temporal variations in bulk water quality in the treatment eﬄuent and at selected locations in the distribution system. Temporal variations should be assessed on both short (hour-to-week) time-scales to detect diurnal patterns and events (Besmer et al., 2014; Nescerecka et al., 2014; Prest et al., 2014; El-Chakhtoura et al., 2015) and long (multi-year) time-scales to detect seasonal changes (Pinto et al., 2014).
• Spatial and temporal investigations of biofilm and sediments should ideally also be included in the design of studies on microbial dynamics in full-scale distribution systems. These two phases are, however, more difficult to sample and therefore not ideal for high frequency monitoring. Sediment sampling can be achieved by distribution networks flushing (Liu et al., 2014). Biofilm sampling can only be performed following pipe extraction from the system (Wingender and Flemming, 2004), e.g., during a pipe replacement by water utilities. Alternatively, biofilm traps/coupons/reactors can be directly connected to the system on long-term basis for representative biofilm formation (Servais et al., 1995; Wingender and Flemming, 2004).

The analysis of water quality should be performed with a combination of methods to analyze microbial and environmental parameters. The following combination of methods is suggested:

• Assessment of bacterial abundance with flow cytometry (e.g., Hammes et al., 2010a; Lautenschlager et al., 2013);
• Assessment of bacterial activity/viability with flow cytometry combined with viability-targeted fluorescent dye and complemented with ATP measurements (e.g., Vital et al., 2012a; Nescerecka et al., 2014);
• Detection of basic shifts in bacterial community composition by analysis samples with flow cytometric fingerprints (Prest et al., 2014);
• In-depth analysis of bacterial community composition with 16S rRNA-gene sequencing methods (e.g., pyrosequencing or Ilumina; Pinto et al., 2014; Roeselers et al., 2015).
• Analysis of environmental parameters, including temperature, pH, conductivity, concentrations of biodegradable organic (e.g., AOC and BDOC) and inorganic nutrients (e.g., phosphate, sulfur, and nitrogen-based compounds such as ammonium and sulfate, methane, and metallic compounds such as iron and manganese). AOC and BDOC tests in this regard are not used as growth predictive methods but rather as measurements of organic content of water, useful for interpretation of data collected with other analysis.

### Predictive Methods for Evaluation of Potential for Biological Stability

Predictive methods are useful to provide supportive information for decision-making of water treatment and/or distribution conditions improvements. Tests should be applied to the treatment eﬄuent and on a selection of distribution locations to evaluate both properties of treated water and how these are affected by distribution conditions. Depending on the question addressed, laboratory tests can include:

• Evaluation of the inherent growth potential of drinking water samples, by direct, untreated incubation of water under controlled laboratory conditions (Gillespie et al., 2014). Incubation conditions can be adapted to be similar to those encountered in distribution systems with low water temperature (e.g., 12°C) and possibly long residence times (e.g., 10 days).
• Identification of growth-limiting nutrients in treated water, by adding a selection of nutrients prior incubation of the treated water in controlled laboratory conditions (Miettinen et al., 1999; Ihssen and Egli, 2004);
• Evaluation of promoting properties of water for biofilm formation, using e.g., the BFR method;
• Evaluation of growth-promoting properties of pathogenic organisms, alone or in competition with the indigenous bacterial community (Vital et al., 2008, 2012b);
• Evaluation of growth-controlling properties of materials in contact with water (e.g., Bucheli-Witschel et al., 2012).

### Applications

The extensive tests proposed can be applied individually or combined depending on targeted information. Below are listed a number of applications.

#### Water Quality Monitoring

Routine monitoring of drinking water quality is essential for surveillance of water quality. A number of chemical/environmental parameters (e.g., pH, conductivity, temperature) are nowadays used for water quality monitoring in real time with on-line apparatus. However, monitoring of microbial aspects remain conservative with the use of cultivation methods for both general water quality (HPC) and hygienically relevant organisms. Disadvantages of cultivation-based methods have been discussed in Section “How is Biological Stability Assessed?”, but essentially, results are only obtained few days after sampling, which does not allow for rapid detection of system failure (e.g., pipe leakage or break) and for immediate corrective actions. Flow cytometry in this regard is an excellent candidate as rapid method for general microbial water quality monitoring, by providing results within 15 min (Hammes et al., 2008; Prest et al., 2013). Moreover, on-line flow cytometric technologies are emerging (Besmer et al., 2014) and one could envision in future real-time monitoring of total and intact bacterial cell counts in treatment eﬄuents and water at several locations in distribution systems. Measurement of total and free ATP concentrations are also to consider for viability assessment and can easily be implemented as routine measurement, as already applied by water utilities in the Netherlands. Collection of large data on water quality in distribution systems in space and time enables description of inherent variations (e.g., daily or seasonal) to each drinking water distribution system under normal conditions and to provide a baseline for detection of abnormal changes (LeChevallier et al., 1996; Besmer et al., 2014). Application of such an approach on long-term would provide sound basis for establishing the degree of acceptable change specific to a given distribution network, or when action is required to safeguard water quality during distribution. In such a case of abnormal change, the continuous control with flow cytometry should be complimented with other methods, such as high-throughput sequencing, or screening for specific pathogenic organisms.

One key advantage of implementing monitoring of microbial parameters in water on a high-frequency basis is the sensitivity for detection of a change in drinking water quality or characteristics. Any change in chemical/environmental property of water would result in a change in the bacterial community (cf. section “A Deeper Look into Microbial Dynamics in Drinking Water”). This change could be detected sensitively by flow cytometry, ATP or 16S rRNA-gene sequencing methods, while other methods for measuring chemical properties of water would not be sufficiently sensitive. As an example, an increase in organic components in water of 1 μg C/L would result in an increase of 10^4^ cells/mL. Flow cytometry enables the counting of bacterial cells down to concentrations of 100 cells/mL. Theoretically, this implies that FCM would enable to quantify bacterial growth occurring from the consumption of 0.01 μg C/L. In comparison, detection limits of AOC methods are usually in the range of 1–10 μg C/L, while the current analytical techniques for DOC measurements have higher detection limits (from 10 μg C/L in the case of high quality apparatus).

#### Improvement of Treatment and Distribution Condition

Combined results obtained from *in situ* and laboratory-scale analyses provide a basis for water treatment and distribution conditions evaluation. The extent of bacterial growth that water can support in bulk water and in biofilm can be assessed using growth potential tests without sample pre-treatment (Gillespie et al., 2014) and the BFR test. Adaptation of the growth potential test is also useful for identification of growth-limiting compounds. The gathered information provide basis for choosing adequate treatment(s) to reduce growth potential of water, by decreasing concentration of growth-limiting compound. Besides, the effect of changing operation conditions of specific treatment (e.g., contact time within biofilters) or of implementation of new treatment steps can be evaluated using the same methods. However, decisions should also be grounded on basis of on-site measurements that evaluate the extent of growth actually occurring within a specific distribution system, based on flow cytometric and ATP measurements.

#### In-depth Research for New Insights in the Biological Stability Concept

The large set of methods proposed can be applied to unravel key knowledge gaps highlighted in previous sections. Particularly, high-throughput 16S rRNA-gene sequencing methods combined with quantitative methods, such as FCM and ATP, offer new opportunities to investigate drinking water ecology related questions (cf. section “How is Biological Stability Assessed?”), including the occurrence of autotrophic growth or the interactions between bulk water, sediments, and biofilms. Similarly, growth potential tests would be of specific value to investigate water limitations in inorganic nutrients. Furthermore, systematic large-scale studies applying the combined methods would provide information related to the establishment of guideline values for a degree of acceptable change in drinking water distribution systems.

## Conclusion

The production and distribution of biological stable drinking water should be a non-negotiable goal for water utilities with the perspective of providing the same water quality to consumers than produced at the treatment facility. This can only be achieved by adequate monitoring and control of microbial processes during water treatment and distribution. Research in the past 30 years has significantly increased knowledge on factors driving changes in microbial water quality during drinking water distribution. These findings have led to implementation and improvement of new water treatment strategies, distribution system designs, and good operation and maintenance practices. However, there are still large knowledge gaps, and a change in microbial water quality is not systematically avoided. Emerging analytical and molecular methods, such as flow cytometry and high-throughput sequencing methods, open new ways for increased understanding of drinking water distribution pipeline ecology. These should be combined and implemented in unified approaches for the study of microbial dynamics in full-scale drinking water distribution systems. Moreover, new perspectives for drinking water quality monitoring are offered by novel analytical methods such as fully automated and on-line flow cytometric analysis.

## Author Contributions

All authors listed, have made substantial, direct and intellectual contribution to the work, and approved it for publication.

## Conflict of Interest Statement

The authors declare that the research was conducted in the absence of any commercial or financial relationships that could be construed as a potential conflict of interest.

## Abbreviations

AOC: assimilable organic carbon
ATP: adenosine-tri-phosphate
BDOC: biodegradable dissolved organic carbon
BFP: biofilm formation potential
BFR: biofilm formation rate
BOM: biodegradable organic matter
BPP: biomass production potential
CFU: colony forming units
DGGE: denaturing gradient gel electrophoresis
DOC: dissolved organic carbon
EPS: extracellular polymeric substances
FCM: flow cytometry
FISH: fluorescence *in situ* hybridization
HPC: heterotrophic plate count
ICC: intact cell concentration
IEX: ion exchange
MF: microfiltration
NF: nanofiltration
NOM: natural organic matter
OTU: operational taxonomic unit
PCR: polymerase chain reaction
PVC: polyvinylchloride
QPCR: quantitative polymerase chain reaction
RO: reverse osmosis
TCC: total cell concentration (Flow cytometry)
TOC: total organic carbon
T-RFLP: terminal restriction fragment length polymorphism
UF: ultrafiltration
VBNC: viable but not culturable
