# Supplementary material for: Biological Stability of Drinking Water: Controlling Factors, Methods, and Challenges
Source: Front Microbiol. 2016 Feb 1;7:45. doi: 10.3389/fmicb.2016.00045 (PMC4740787; doi:10.3389/fmicb.2016.00045)
Supplement: Supplementary file 1 [file Table_1.PDF]

Table S1. Selection of relevant publications on biological stability of drinking water and/or on microbial growth in distribution systems, with an overview of study focus, considered microbial growth controlling factors, study approach and methods, and if applicable, proposed guideline values and definition for biological stability (BS).

**Authors**	**Focus**					**Considered microbial growth controlling factors**		**Approach and methods**			**Guideline value? Definition BS?**
	Study focus	Temporal/ spatial variations?	Residual disinfectant?	Type of organisms	Organisms location	nutrients	Other factors	Approach microbial study	Microbial analysis	Other methods	
Rittmann, Snoeyink, 1984	Treated water characteristics	-	General	All organisms, focus on heterotrophic + autotrophic bacteria	Suspended	All, focus on NH^{4+}, BOM, Mn	-	-	-	-	No guideline value<br>Definition Nr. 1
Maul et al., 1985/ Kerneis et al., 1995	Treated water characteristics + distribution effect	Spatial + temporal (seasonal)	Yes	Heterotrophic bacteria	Suspended	-/ BOM	Disinfectant concentration, residence time, temperature	Assessment	HPC (R2A)	-/BDOC	No guideline value<br>No definition
Van der Kooij et al., 1992	Treated water characteristics + distribution effect	Spatial + temporal (seasonal)	No	Heterotrophic bacteria	Suspended	BOM	Residence time, distance	Assessment	HPC(PCA), total coliforms, total ATP	AOC[1]	AOC < 10 µg Ac-C eq/L<br>No definition
Servais et al., 1992, 1995	Treated water characteristics + distribution effect	Spatial + temporal (seasonal)	Yes	All bacteria, focus on heterotrophic bacteria	Suspended + fixed (biofilms)	BOM	Disinfectant concentration, temperature, pipe diameter	Assessment, prediction*	TDC, ^{3}H thymidine incorporation	BDOC Models*	BDOC < 150 µg/l<br>No definition
LeChevallier 1992, 1996	Treated water characteristics + distribution effect	Spatial + temporal (seasonal)	Yes	Heterotrophic bacteria, focus on coliforms	Suspended	BOM	Disinfectant concentration and type temperature, rainfall	Assessment	HPC (R2A), total coliforms	AOC[1,2]	AOC < 50 µg Ac-C eq/L, Temp <15°C (1991), AOC < 100 µg Ac-C eq/L (1992)<br>No definition
Volk, Joret, 1994	Treated water characteristics + distribution effect	Spatial + temporal (seasonal)	Yes	All bacteria, focus on coliforms	Suspended	BOM	Disinfectant concentration, temperature	Assessment, prediction*	Total coliforms, TDC	BDOC, Models*	No definition<br>Temp < 15°C<br>BDOC < 0.15 mg/L<br>Free Chlorine>0.10 mg/L
Miettinen et al., 1997	Treated water characteristics	Spatial	General	Heterotrophic bacteria	Suspended	All, focus on MAP	-	Prediction	HPC (R2A)	AOC[3] (nutrient addition)	No guideline value<br>No definition

Prévost et al., 1998	Treated water characteristics + distribution effect	Spatial + temporal (seasonal)	Yes	All bacteria, focus on heterotrophic bacteria	Suspended	BOM	Disinfectant concentration, residence time, pipe surface/ volume ratio, temperature	Assessment	HPC, TDC, DVC, ^{3}H thymidine incorporation	BDOC	No guideline value No definition
Sibile, 1998a	Treated water characteristics + distribution effect	Spatial	General	Heterotrophic bacteria, protozoa	Suspended + fixed (biofilms)	BOM	Protozoa, sediments, disinfectant concentration, residence time, temperature	-	-	-	No guideline value Definition Nr. 2
Van der Kooij, 2000, 2003	Treated water characteristics + distribution effect	Spatial	General	Heterotrophic bacteria, incl. undesirable bacteria	Suspended + fixed (biofilms, sediments)	BOM	Pipe material temperature, residence time, flow velocity	Assesment, prediction*	HPC (R2A),total ATP, PCR, FISH	AOC[1]* BFR* BFP* BPP* Models*	AOC < 10 µg Ac-C eq/L BFR < 10 pg ATP/cm^{2}.d Definition Nr. 3
Niquette et al., 2001	Treated water characteristics + distribution effect	Spatial	Yes	Heterotrophic bacteria	Suspended + fixed (biofilms)	BOM	Disinfectant concentration, residence time, temperature	Assessment	TDC, 3H thymidine incorporation	BDOC	BDOC < 0.25 mg C/L No definition
Lehtola et al., 2006	Treated water characteristics + distribution effect	-	Yes	All bacteria, focus on heterotrophic bacteria	Suspended+ fixed (biofilms, sediments)	BOM, MAP, Iron, copper	Sediments, pipe material, flow velocity, temperature	Assessment	TDC, HPC (R2A), total coliforms, RT-PCR (viral analysis)	TOC, AOC[3], MAP	No guideline value No definition
Pepper et al., 2004	Treated water + distribution effect + household effect	Spatial	Yes	Heterotrophic + autotrophic bacteria	Suspended	-	-	Assessment	HPC (R2A), identification of specific bacterial groups	-	No guideline value No definition
Batte et al., 2006,	Treated water + distribution effect	Spatial + temporal (seasonal)	Yes	Heterotrophic + autotrophic bacteria, focus on bacterial indicators	Suspended+ fixed (biofilms)	BOM,	Disinfectant concentration, residence time, temperature	Assessment	HPC (R2A), total coliforms, enterococci	BDOC	No guideline value No definition

Srinivasan, Harrington, 2007	Treated water	Spatial	Yes	Heterotrophic + autotrophic bacteria	Suspended	AOC, NH^{4+}	Disinfectant concentration and type	Prediction*	HPC	Models*	No guideline value No definition
Manuel et al., 2010	Treated water + distribution effect + household effect	Temporal	Yes	All bacteria, focus on heterotrophic bacteria	Suspended + fixed (biofilms)	BOM	Stagnation, unsteady flow rates	Assessment	TDC, HPC (R2A), FISH	TOC	No guideline value No definition
Lautenschlager et al., 2010	Household effect	Temporal	No	All bacteria	Suspended	-	Stagnation: residence time, temperature,	Assessment	TCC and ICC, total/free ATP, HPC, DGGE	AOC[4]	No guideline value No definition
Lautenschlager al., 2013	Treated water + distribution effect	Spatial + temporal	No	All bacteria	Suspended	BOM	Residence time, temperature	Assessment	TCC and ICC, HPC, total/free ATP, DGGE, Pyro-sequencing	AOC[4]	No guideline value Definition Nr. 4
Nescerecka 2014	Treated water + distribution effect	Spatial + temporal (short-term)	Yes	All bacteria	Suspended	BOM	Disinfectant concentration, residence time	Assessment	TCC and ICC, total/free ATP, HPC (PCA)	DOC, AOC[1]	No guideline value No definition
Pinto et al., 2012, 2014	Treated water + distribution effect	Spatial + temporal (seasonal)	Yes	All bacteria	Suspended	BOM, NH^{4+}, PO_4^{3-}, SO_4^{2-}	Distance, temperature, pH	Assessment	16S rRNA Pyro-sequencing	-	No guideline value No definition
Liu et al., 2013a, b, 2014	Treated water + distribution effect	Spatial + temporal (seasonal)	No	All bacteria	Suspended+ fixed (biofilms, sediments, suspended solids)	BOM	Pipe material, disinfectant concentration and type, distance, temperature	Assessment	HPC (R2A) TCC total/free ATP, Aeromonas 16S rRNA Pyro-sequencing	-	No guideline value No definition
Wang et al., 2014	Treated water + distribution effect	Spatial	Yes	All bacteria, + eukaryotes, focus on opportunistic pathogens	Fixed (biofilms)	-	Pipe material disinfection concentration and type, residence time	Assessment	16S rRNA Pyro-sequencing, T-RFLP	-	No guideline value No definition

* indicates the tools used as predicative methods.

Abbreviations: AOC: assimilable organic carbon; ATP: adenosine-tri-phosphate; BDOC: biodegradable dissolved organic carbon; BFP: biofilm formation potential; BFR: biofilm formation rate; BPP: Biomass production potential; DGGE: Denaturing gradient gel electrophoresis; DOC: dissolved organic carbon; DVC: direct viable count (epifluorescence microscopy, staining with CTC); HPC: heterotrophic plate count (the medium is indicated between brackets); FISH: fluorescence in situ hybridization; ICC: intact cell concentration (Flow cytometry); MAP: microbially available phosphate; PCR: polymerase chain reaction; RT-PCR: Reverse transcription polymerase chain reaction; TCC: total cell concentration (Flow cytometry); TDC: total direct counts (epifluorescence microscopy, staining with acridine orange or DAPI); TOC: total organic carbon; T-RFLP: Terminal restriction fragment length polymorphism

AOC methods

1. Van der Kooij et al., 1982, 1985: Sample autoclaving, inoculation with pure strains P17 and NOx, bacterial growth assessment by plate counting
2. LeChevallier et al., 1993: Sample autoclaving, inoculation with pure strains P17 and NOx, bacterial growth assessment by ATP measurement
3. Miettinen et al., 1999 : Sample autoclaving, inoculation with pure strains P17 and NOx, nutrients addition (mixture of several elements needed for bacterial growth) bacterial growth assessment by plate counting
4. Hammes and Egli, 2005 : sample filtration, inoculation with natural bacterial community, bacterial growth assessment by flow cytometry.

Definitions:

1. “A biological stable water does not support the growth of microorganisms to a significant extent, whereas an unstable water supports high numbers of microbes in distribution systems if sufficient disinfectant is not used”.
2. “A biological stable water is a water for which bacterial growth, organic matter consumption and predation are not significant”.
3. “Biostability is defined as the inability of water or a material in contact with water to support microbial growth in the absence of a disinfectant”.
4. “Biological stability would imply no changes occurring in the concentrations and composition of the microbial community in the water during distribution”.
